# The Pittsburgh Sleep Quality Index: Reliability, Factor Structure, and Related Clinical Factors among Children, Adolescents, and Young Adults with Chronic Pain

**DOI:** 10.1155/2021/5546484

**Published:** 2021-04-26

**Authors:** Cynthia L. Larche, Isabelle Plante, Mathieu Roy, Pablo M. Ingelmo, Catherine E. Ferland

**Affiliations:** ^1^Shriners Hospitals for Children–Canada, 1003, Boul. Décarie, Montréal, QC, Canada H4A 0A9; ^2^Département de didactique, Université du Québec à Montréal, C.P. 8888, Succursale Centre-Ville, Montréal, QC, Canada H3C 3P8; ^3^The Alan Edwards Centre for Research on Pain, 740 Dr. Penfield Avenue, Montréal, QC, Canada H3A 0G1; ^4^Department of Psychology, McGill University, 2001 McGill College, Montréal, QC, Canada H3A 1G1; ^5^Chronic Pain Services, Montreal Children's Hospital, 1001 Boulevard Décarie, Montréal, QC, Canada H4A 3J1; ^6^Department of Anesthesia, McGill University Health Centre, 1001 Boul. Décarie, Montréal, QC, Canada H4A 3J1; ^7^The Research Institute of the McGill University Health Centre, 1001 Boul. Décarie, Montréal, QC, Canada H4A 3J1

## Abstract

This study is aimed at assessing the psychometric properties and the factorial structure of the Pittsburgh Sleep Quality Index (PSQI) in a clinical sample of children, adolescents, and young adults with chronic pain. Data of 482 participants (aged 8-21 years) from two crosssectional studies and a chronic pain services outpatient clinic were analyzed. Exploratory and confirmatory factor analysis and reliability analysis of PSQI component scores were performed. Relationships between the PSQI global score and various clinical measures were investigated to assess external validity. The findings exhibit the reliability and validity of a single-factor model of the PSQI in a clinical sample of youth with chronic pain and support the relationship in this specific population between poor sleep quality and important clinical measures of well-being. These results support an informed decision regarding its use with this specific population and underscore the clinical relevance of assessing sleep quality.

## 1. Introduction

Chronic pain is a prevalent issue in children, adolescents, and young adults (CAYA), often presenting with important problems in daily functioning [1]. Young people with chronic pain (that has persisted for more than three months) commonly experience poor sleep quality [2]. Occurring during critical stages of cognitive development, poor sleep quality can have far-reaching negative consequences on personal relationships, emotional state, and school performance [3]. Moreover, sleep quality in this specific population has been shown to be associated with age [4], pain intensity [5], functional disability [6], and symptoms of anxiety and depression [5–9]. These findings suggest that the impact of poor sleep quality on CAYA living with chronic pain is significant.

To improve the management of chronic pain in young patients, it is of paramount importance that clinicians identify poor sleep quality. With patients who can self-report, healthcare providers commonly assess sleep quality by administrating the Pittsburgh Sleep Quality Index (PSQI), one of the most frequently used general measures of sleep quality in clinical and research settings [10]. The PSQI is a 19-item questionnaire that was developed and initially validated in adults by Buysse et al. [11] to assess sleep quality over the previous month, yielding a global score that facilitates score comparison between groups or individuals over time.

A systematic review and meta-analysis of 37 psychometric studies of the PSQI in both clinical and nonclinical adult and pediatric samples reported good internal consistency and convergent validity [10]. However, the studies with samples of young people supported different factorial structure models of the PSQI. For instance, Raniti et al. [7] performed exploratory and confirmatory factor analyses (EFA and CFA, respectively) of the PSQI in a community-based sample of adolescents, and their results validated a single-factor structure. The single-factor model was also supported by de la Vega et al. [8] with a CFA in a community-based sample of adolescents and young adults. In contrast, the EFA conducted by Benhayon et al. [9] using a clinical sample of children and adolescents with comorbid Crohn's disease and depression validated a two-factor structure, which Passos et al. [12] replicated with a CFA using a community-based sample of CAYA. Furthermore, the final models in all but one [7] of the CAYA studies found that model fit was improved with the removal of the “Use of sleeping medication” component. Overall, the discrepancy between the supported models may be attributable to group differences (e.g., age, disease, and culture). A review of pediatric sleep tools by Sen and Spruyt [13] supported the need for more psychometric studies within specific populations. Therefore, a validation of the PSQI among CAYA with chronic pain was warranted and would support its use as a clinical tool with this population.

The principal aim of the present study was to examine the psychometric properties of the PSQI. Specifically, the study intended to validate the single-factor structure of the tool and to determine the reliability of the PSQI global score in a clinical sample of CAYA with chronic pain. It was predicted that the findings would support the original single-factor model, specifically that the results of an EFA conducted would be replicated in a CFA. Consistent with previously reported coefficient values, it was hypothesized that the internal consistency of the PSQI components would be acceptable (Cronbach′s *α* > 0.70) [10].

Aside from evaluating the psychometric properties of the PSQI, the study also examined the associations between the PSQI global score of young chronic pain patients and clinical variables, including pain characteristics, functional disability, and anxiety and depression. In doing so, the study would provide support for the external validity of the PSQI for use with this specific population.

## 2. Materials and Methods

### 2.1. Participants

The data in the present analyses were obtained from two crosssectional studies conducted at the Shriners Hospitals for Children–Canada and from the database of the Chronic Pain Service (CPS) of the Montreal Children's Hospital. Ethics approval was obtained prior to the beginning of the studies from the Institutional Review Board of the Faculty of Medicine of McGill University (A11-M62-15B; A09-M17-17B) and the Research Ethics Board of the McGill University Health Center (2019-4887). The studies were carried out in accordance with the principles of the Declaration of Helsinki. Prior to the beginning of each study, written consent was obtained from participants 14 years of age or older. For participants less than 14 years old, parental consent and participant assent were obtained.

The data included in the present analyses were collected from January 2016 to January 2020. Eligibility criteria were that patients had chronic pain confirmed by a physician and that they had the ability to read and write in English or French. Participants completed self-reported questionnaires on the day of the study or CPS visit. Patients were excluded if they were unable to complete self-report measures. Patients were excluded from the analyses if any PSQI items were missing.

The final sample included 482 participants (8-21 years old; 391 females and 91 males). The diagnoses included chronic secondary musculoskeletal pain (64.52%), chronic primary pain (23.24%), chronic postsurgical or posttraumatic pain (9.13%), chronic neuropathic pain (2.07%), chronic secondary visceral pain (0.41%), chronic secondary headache or orofacial pain (0.41%), and chronic cancer-related pain (0.21%).

### 2.2. Measures

#### 2.2.1. Demographics and Pain Characteristics

Clinical information gathered included age, gender, pain diagnosis, duration of pain (3-6 months, 6-12 months, or>12 months), and painful episode duration (intermittent or constant). Participants completed the Adolescent Pediatric Pain Tool [14], a pain quality tool which was used to determine whether they experienced one or more pain sites. Participants were also asked to report their current overall pain intensity using a scale of 0-10, where zero was no pain and 10 was the worst pain imaginable.

#### 2.2.2. Quality of Sleep

Participants' quality of sleep was measured with the PSQI [11]. The original version of the PSQI consists of 19 items [11]. As the scoring of the tool only includes self-rated items, the 19^th^ item was excluded from this study. The first four items are in a free-response format and assess sleep duration. The remaining items are related to sleep disturbances and daytime dysfunction. On a 4-point Likert scale, participants indicate the frequency of each problem (0 = not during the past month, 1 = less than once a week, 2 = once or twice a week, 3 = three or more times a week) and their sleep quality overall (0 = very good, 1 = fairly good, 2 = fairly bad, 3 = very bad). The items are scored nonlinearly to generate seven component scores: subjective sleep quality, sleep latency, sleep duration, habitual sleep efficiency, sleep disturbances, use of sleeping medication, and daytime dysfunction. Each component is scored 0-3, and the sum of the components yields a global score (0-21), where a higher score indicates greater difficulty in all component areas. Poor sleep is identified with a global score greater than five [11].

#### 2.2.3. Physical Functioning and Disability

Participants' physical everyday functioning was assessed with the Functional Disability Inventory (FDI), a 15-item questionnaire. The tool was originally developed for use with children and adolescents with chronic abdominal pain [15]. Each item is rated on a five-point Likert scale (0 = no trouble, 1 = a little trouble, 2 = some trouble, 3 = a lot of trouble, 4 = impossible). The total score ranges 0-60, where higher scores indicate greater activity limitations during the past two weeks. Clinical reference points for the FDI have been established in the pediatric chronic pain population (no/minimal disability (0-12), moderate disability (13-29), and severe disability (≥30)) [16].

#### 2.2.4. Symptoms of Anxiety and Depression

Participants' self-reported symptoms corresponding to anxiety disorders and depression, as per the Diagnostic and Statistical Manual of Mental Disorders IV, were evaluated using the 47-item Revised Children's Anxiety and Depression Scale (RCADS) [17]. The RCADS has shown good psychometric properties in a clinical sample of children and adolescents and has demonstrated strong clinical utility to screen for diagnoses and track clinical changes [18]. The tool yields a Total Anxiety Scale (anxiety subscales) and a Total Internalizing Scale (all subscales). Items are rated on a four-point Likert scale (0 = never, 1 = sometimes, 2 = often, 3 = always). The rater must sum the scores of the items subsumed within each scale to determine the corresponding *T*-score (below clinical threshold (<65), borderline (65-70), and above clinical threshold (>70)).

### 2.3. Statistical Analyses

The PSQI component and global scores were calculated according to standard scoring procedures [11]. Descriptive statistics and two-tailed Pearson correlations between the component scores were computed using the Statistical Package for Social Science (SPSS) (Version 26). For the reliability analysis, Cronbach's alpha was calculated using the components, with *α* > .70 indicating acceptable internal consistency [19].

Bartlett's test of sphericity was significant (*χ*^2^ (21) = 804.89, *p* < .001), which indicated that the error correlations between the items are significantly different from zero and supported the relevance of performing principal component analysis (PCA) [19]. In addition, results of the Kaiser-Meyer-Olkin (KMO) measure provided a value of 0.740, which corresponds to a good sampling adequacy [19]. An EFA, also performed with SPSS, was used to test whether the components conformed to the single-factor structural model. The EFA was conducted using PCA and maximum likelihood (ML) factor extraction methods. The ML method was used for comparison as it has been used in previous PSQI studies [7, 20]. As the factors are correlated, a direct oblimin rotation of factors with Kaiser normalization was used [21]. During the initial analysis, factors with an eigenvalue > 1 were retained in the model per Kaiser's criterion [22]. As recommended, variables with factor loadings > .3 were retained within the factors [23]. Factors were considered unreliable if they retained less than three variables that were not strongly correlated (*r* < .70) [24]. Using the a priori criterion, a single-factor extraction was also performed to evaluate the original factor structure [23].

A CFA was performed with SPSS Amos (Version 26) to examine whether the models supported by the EFA could be further confirmed. Several model fit indices were used to assess the adequacy of the model: the chi-square test (*χ*^2^) and its ratio with the degree of freedom (*χ*^2^/df), the Root Mean Square Error of Approximation (RMSEA), and the Comparative Fit Index (CFI) [25, 26]. The chi-square evaluates the level of discrepancy between the fitted and sample covariance, with nonsignificance indicating an acceptable model. However, the chi-square test is known for its sensitivity to sample size [27]. Especially in large samples, significant results are often found even if the model should not be rejected [28]. Therefore, other goodness-of-fit indicators were also considered. A reasonable model fit is indicated when the *χ*^2^/df ratio is <5 [29]. For the RMSEA, values < .05 suggest an excellent model fit, whereas values .05-.08 indicate a good fit [30]. For the CFI, cut-off values close to or over .95 indicate an acceptable fit [31]. The Akaike Information Criterion (AIC) penalizes for overparameterization and was used to compare the goodness-of-fit of the models, with a lower value indicating better fit [32, 33]. If the goodness-of-fit indices do not reach cut-offs, the model can be modified to be more parsimonious, on the condition that the added paths are theoretically grounded [34].

Descriptive statistics were used to assess demographic and outcome measures. Two-tailed Pearson correlations were used to investigate the relationship between sleep quality and age, functional disability, symptoms of anxiety and depression, as well as pain intensity. A two-way independent sample analysis of variance (ANOVA) was performed to assess whether sleep quality differed as a function of gender and chronic pain duration. One-way independent group ANOVAs were performed to assess whether sleep quality differed as a function of painful episode duration, reported pain during movement, and the number of pain sites.

## 3. Results

### 3.1. Reliability and Validity of the PSQI

#### 3.1.1. Descriptive Statistics and Correlations

Mean scores and standard deviations of the components and the correlation between each component (Table 1) were examined. The mean PSQI global score was 7.67 ± 3.81. Correlations between the PSQI components were all statistically significant. Nearly one-third of participants (32.16%) reported taking sleeping medication in the last month, with 14.94% indicating a frequency of three or more times per week.

#### 3.1.2. Reliability Analysis

Cronbach's *α* for the seven component scores that comprise the PSQI global score was 0.74, which suggests good internal consistency (Table 1). Staying true to the original single-factor model, the “Use of sleeping medication” component was retained because its removal would lead to a negligible improvement to Cronbach *α* (0.75).

#### 3.1.3. Exploratory Factor Analysis

The initial EFA results indicated a two-factor model (Table 2). The PCA and ML extractions yielded similar factor loadings, which all met the cut-off criterion. The factors' eigenvalues of 2.88 and 1.12 cumulatively explained 57.03% of the variance. However, one of the factors contained only two component variables (“Sleep duration” and “Habitual sleep efficiency”), which were not strongly correlated, and thus that factor proved to be unreliable. In contrast, the PCA and ML single-factor extraction produced a factor that explained 41.07% of the variance. Both methods yielded similar factor loadings (Table 2) that met the cut-off criterion, and thus, the model was considered final. The results of the EFA supported a single-factor model, including all PSQI components.

#### 3.1.4. Confirmatory Factor Analysis

**Figure 1:**
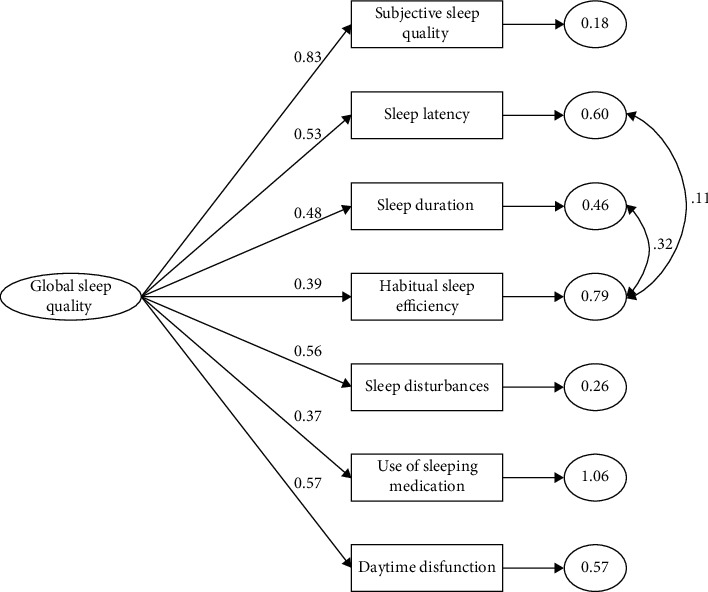
Final Pittsburgh Sleep Quality Index single-factor model, with all maximum likelihood estimates (standardized estimates) and error terms significant at *p* < .001.

Based on the results of the reliability and EFA, all PSQI components were included in a CFA (Table 3). The initial model was not accepted as the *χ*^2^, *χ*^2^/df, RMSEA, and CFI did not reach the prespecified model fit criteria. Based on the modification indices, covariance between the residuals of the PSQI components “Sleep Latency” and “Habitual Sleep Efficiency” and between the components “Sleep Duration” and “Habitual Sleep Efficiency” was added to the model. These pairs of PSQI components have a strong content overlap, as sleep latency affects sleep duration and vice versa, and both components impact the calculation of the sleep efficiency component score. Although the results of the new model showed a significant *χ*^2^ value, the AIC value decreased, and an adequate fit of the criteria for the *χ*^2^/df, RMSEA, and CFI was achieved; therefore, the model was considered final (Figure 1).

### 3.2. External Validity of the PSQI

Means and standard deviations for the outcome variables were examined (Table 4). The analyses yielded moderately significant correlations between poor sleep quality and functional disability (*r* = .52, *p* < .001) and anxiety and depression (*r* = .43, *p* < .001), as well as weak but significant correlations with pain intensity (*r* = .23, *p* < .001) and participant age (*r* = .18, *p* < .001).

There was no significant difference between genders (*F*(1,476) = 1.335, *p* = 0.248, *η*^2^ = 0.003) in sleep quality, demonstrating that PSQI global scores between females and males did not significantly differ. Similarly, there was no significant difference in sleep quality as a function of chronic pain duration (*F*(2,476) = 0.099, *p* = 0.905, *η*^2^ = 0.000). These results reveal that there were no significant differences in PSQI global scores between participants who had experienced pain for 3-6 months, 6-12 months, or >12 months.

Results showed that sleep quality did vary as a function of the duration of painful episodes (*F*(1,394) = 5.38, *p* = 0.021, *η*^2^ = 0.013), showing that significantly higher PSQI global scores were obtained by participants who reported constant pain compared to those who reported intermittent pain. Similarly, participants who reported pain during movement obtained significantly higher PSQI global scores (*F*(1,393) = 6.82, *p* = 0.009, *η*^2^ = 0.017) compared to those who reported no pain during movement. Likewise, sleep quality differed according to the number of pain sites (*F*(1,465) = 19.14, *p* < .001, *η*^2^ = 0.04), demonstrating that participants who reported more than one pain site obtained significantly higher PSQI global scores compared to those who reported only one.

## 4. Discussion

Results showed that the EFA supported a single-factor model, a result that was further confirmed by a CFA. Internal consistency was acceptable. External validity was demonstrated as the results showed that PSQI scores correlated with other symptoms such as age, pain intensity, functional disability, and symptoms of anxiety and depression. Sleep quality varied according to the duration of painful episodes, the presence of pain during movement, and the number of pain sites. These findings further support the validity of the PSQI for a clinical sample of CAYA with chronic pain.

The evaluation of the psychometric properties of the PSQI in this population showed that the mean PSQI global score and mean PSQI component scores (7.67 ± 3.81; 0.53-1.82) were considerably higher than in a community-based sample of adolescents (6.36 ± 3.22; 0.24-1.54) [7]. These results indicate that this particular clinical sample generally experienced worse sleep quality than did healthy adolescents. The correlations between PSQI components were alike [7] and suggest that the dimensions of sleep quality are comparable across populations of similar age, regardless of whether they are clinical or nonclinical samples. The reliability analyses yielded an acceptable value of internal consistency, despite that other authors have argued that the psychometric properties of the PSQI would be improved by excluding the sleep medication component [8, 12]. Although the removal of the component would have improved the value of Cronbach's *α*, the improvement would be minimal. This component also met the variable retention criterion in the EFA. Furthermore, as nearly one-third of the participants reported taking sleep medication in the past month and 15% of the sample reported a frequency of three or more times per week, the contribution of this component score to the PSQI global score with this specific population was substantial. The component score may additionally serve as an indicator supporting the clinician's decision for treatment. This being said, the factor analyses produced an interesting trend, as the two-factor model initially found with the EFA included a factor onto which loaded the components “Sleep duration” and “Habitual sleep efficiency.” Although the CFA supported a final, single-factor model, it was achieved by allowing covariance between the residuals of those same PSQI components as well as between those of “Sleep latency” and “Habitual sleep efficiency.” Despite the demonstrated links in the EFA and CFA, it could be argued that neither pair of components represented separate latent constructs as the relationship between the components could be attributable to an overlap in the items required to compute the component scores. The variation in the overall factor structure model of the PSQI supported across studies of samples of CAYA [7–9, 12] may be due to differences in the range of sleep disorders experienced by young people. Although the majority of the studies included in the systematic review by Manzar et al. [35] supported a two-factor structure of the PSQI, the authors accentuated that the heterogeneity of the studies' findings may be attributed in part to differences in the studies' reported methodology. The reviewed studies varied significantly with regard to their factor analyses, such as factor extraction methods, variable retention criteria, and goodness-of-fit indices [35]. For example, some studies retained factors onto which loaded less than three variables, despite best practice recommendations suggesting that such factors are considered unreliable [24]. Overall, the results of the current study extend those of Raniti et al. [7] among a community-based sample of adolescents and provide empirical evidence of the reliability and validity of a single-factor structure of the PSQI for clinical use with CAYA with chronic pain.

The present analyses yielded many significant associations between clinical variables and the PSQI global score. In this sample, older patients experienced significantly worse sleep quality, whereas others did not substantiate the effect of age [9]. As sleep duration tends to decrease throughout adolescence [36], the discrepancy between the findings may be because the present sample included older participants. Moreover, the results showed that sleep quality did not vary based on gender. The sample was 81.1% female, and such a significant representation, albeit typical of chronic pain populations [1], could have masked an underlying gender difference. The results lend additional support to previous studies of the PSQI, which found that sleep quality was significantly associated with pain intensity [5, 37], functional disability [37], and symptoms of anxiety and depression [7–9]. Sleep quality amongst participants did not differ in accordance with how many months they had experienced chronic pain; however, it did vary as a function of painful episode duration, presence of pain during movement, and the number of pain sites experienced. Overall, the results supported the external validity of associations between an array of concerning factors and sleep quality in CAYA with chronic pain and the complex interplay between the variables denoted the importance of treating sleep problems in this clinical population. Future research should evaluate potential predictors of sleep quality by using diaries to document behaviours that might be related to sleep quality, such as the delay between screen time and sleep, caffeine consumption, the practice of yoga, and other mindfulness interventions. Data regarding these habits could also be included in structural equation modeling to provide an empirical validation of the relationship between these behaviours and sleep quality.

Despite the overall contribution of the study to the field, the research also has some limitations. The subjective nature of the self-report measures inevitably left room for affective and interpretational influence. As this was a crosssectional study, test-retest reliability could not be evaluated. Criterion validity of the PSQI as a measure of sleep quality could not be evaluated as there is no gold standard in self-reported sleep quality assessment tools [38]. Moreover, known-group validity could not be assessed for a lack of a healthy control group. Lastly, the cut-off score of the PSQI was not investigated in the present analyses, nor was it assessed by other studies of samples of CAYA. Future research should investigate whether the PSQI global score is better represented by a categorical or continuous construct [10].

## Figures and Tables

**Table 1:** Descriptive statistics and internal consistency (Cronbach's *α*) of the PSQI components and correlations among them.

			Pearson's correlation (*r*)					
PSQI components	Mean (SD)	Cronbach's *α* if item deleted	1	2	3	4	5	6
1. Subjective sleep quality	1.38 (0.74)	0.67	—					
2. Sleep latency	1.82 (0.91)	0.70	.44^∗∗∗^	—				
3. Sleep duration	0.53 (0.77)	0.69	.41^∗∗∗^	.27^∗∗∗^	—			
4. Habitual sleep efficiency	0.64 (0.97)	0.70	.33^∗∗∗^	.33^∗∗∗^	.62^∗∗∗^	—		
5. Sleep disturbances	1.37 (0.61)	0.71	.47^∗∗∗^	.26^∗∗∗^	.22^∗∗∗^	.21^∗∗∗^	—	
6. Use of sleeping medication	0.68 (1.11)	0.75	.31^∗∗∗^	.30^∗∗∗^	.11^∗^	.18^∗∗∗^	.17^∗∗∗^	—
7. Daytime dysfunction	1.25 (0.92)	0.71	.45^∗∗∗^	.24^∗∗∗^	.32^∗∗∗^	.17^∗∗∗^	.40^∗∗∗^	.21^∗∗∗^

Pittsburgh Sleep Quality Index; correlation significance: ^∗^*p* < 0.05, ^∗∗∗^*p* < 0.001 (2-tailed).

Cronbach's *α* for all seven components is 0.74.

**Table 2:** Factor pattern matrix for the PSQI two-factor solution and factor matrix for the PSQI single-factor solution, comparing two extraction methods.

PSQI components	PCA extraction		ML extraction	
PSQI two-factor solution	Factor 1	Factor 2	Factor 1	Factor 2
1. Subjective sleep quality	**0.72**	-0.21	**0.81**	0.01
2. Sleep latency	**0.51**	-0.25	**0.44**	0.16
3. Sleep duration	0.04	**-0.87**	0.23	**0.54**
4. Habitual sleep efficiency	-0.03	**-0.91**	-0.09	**1.04**
5. Sleep disturbances	**0.74**	0.06	**0.59**	-0.03
6. Use of sleeping medication	**0.59**	0.09	**0.35**	0.04
7. Daytime dysfunction	**0.72**	0.02	**0.62**	-0.08
% of total variance	41.07	15.96	21.38	24.49
Interfactor correlation	-0.35		0.47	
PSQI single-factor solution	Factor 1		Factor 1	
1. Subjective sleep quality	**0.79**		**0.77**	
2. Sleep latency	**0.63**		**0.55**	
3. Sleep duration	**0.68**		**0.58**	
4. Habitual sleep efficiency	**0.65**		**0.53**	
5. Sleep disturbances	**0.61**		**0.54**	
6. Use of sleeping medication	**0.46**		**0.37**	
7. Daytime dysfunction	**0.62**		**0.55**	
% of total variance	41.07		31.95	

Pittsburgh Sleep Quality Index, principal component analysis, and maximum likelihood. Factor loadings > .30 are in bold and were retained in the factor.

**Table 3:** Indices of fit for initial and final PSQI single-factor models.

Model	*χ* ^2^ (df)	*χ* ^2^/df	RMSEA	CFI	AIC
Initial model	196.02 (14)	14	0.16	0.77	238.02
Final model	42.03 (12)	3.50	0.07	0.96	88.03

Pittsburgh Sleep Quality Index, chi-squared and degrees of freedom, chi-square and its ratio with degrees of freedom, Root Mean Square Error of Approximation, Comparative Fit Index, and Akaike Information Criterion.

**Table 4:** Descriptive statistics of patient characteristics.

Patient characteristics	Total patient sample	PSQI global score	Significance (*p* value)
Demographics			
Age, years	15.27 (2.11)	—	<.001
Gender, *n* (%)		—	.248
Female	391 (81.12)	7.80 (3.84)	
Male	91 (18.88)	7.12 (3.68)	
Outcome measures			
FDI total score	15.95 (10.35)	—	<.001
RCADS total internalization *T*-score	49.18 (13.92)	—	<.001
Pain intensity	3.74 (2.72)	—	<.001
Duration of chronic pain, *n* (%)			.905
3-6 months	44 (9.12)	8.11 (3.62)	
6-12 months	84 (17.43)	7.26 (3.74)	
>12 months	354 (73.44)	7.71 (3.86)	
Painful episode duration, *n* (%)			.021
Constant pain	209 (52.78)	7.77 (3.73)	
Intermittent pain	187 (47.22)	6.95 (3.26)	
Reported pain during movement, *n* (%)			.009
Reported no pain during movement	45 (11.39)	6.09 (2.69)	
Reported pain during movement	350 (88.61)	7.54 (3.60)	
Number of pain sites, *n* (%)			<.001
One pain site	218 (46.68)	6.83 (3.32)	
More than one pain site	249 (53.32)	8.33 (4.03)	

Pittsburgh Sleep Quality Index, Functional Disability Inventory, and Revised Children's Anxiety and Depression Scale. Data are presented as mean ± SD in parenthesis unless otherwise stated.

## Data Availability

The data used to support the findings of this study have not been made available as they are the property of the Shriners Hospitals for Children–Canada.

## References

[1] King S., Chambers C. T., Huguet A. (2011). The epidemiology of chronic pain in children and adolescents revisited: a systematic review. *Pain*.

[2] Wojtowicz A. A., Banez G. A. (2015). Adolescents with chronic pain and associated functional disability: a descriptive analysis. *Journal of Child Health Care*.

[3] Owens J., ADOLESCENT SLEEP WORKING GROUP, COMMITTEE ON ADOLESCENCE (2014). Insufficient sleep in adolescents and young adults: an update on causes and consequences. *Pediatrics*.

[4] Pavlova M., Kopala-Sibley D. C., Nania C. (2020). Sleep disturbance underlies the co-occurrence of trauma and pediatric chronic pain: a longitudinal examination. *Pain*.

[5] Pavlova M., Ference J., Hancock M., Noel M. (2017). Disentangling the sleep-pain relationship in pediatric chronic pain: the mediating role of internalizing mental health symptoms. *Pain Research & Management*.

[6] Palermo T. M., Kiska R. (2005). Subjective sleep disturbances in adolescents with chronic pain: relationship to daily functioning and quality of life. *The Journal of Pain*.

[7] Raniti M. B., Waloszek J. M., Schwartz O., Allen N. B., Trinder J. (2018). Factor structure and psychometric properties of the Pittsburgh Sleep Quality Index in community-based adolescents. *Sleep*.

[8] de la Vega R., Tomé-Pires C., Solé E. (2015). The Pittsburgh Sleep Quality Index: validity and factor structure in young people. *Psychological Assessment*.

[9] Benhayon D., Youk A., McCarthy F. N. (2013). Characterization of relations among sleep, inflammation, and psychiatric dysfunction in depressed youth with Crohn disease. *Journal of Pediatric Gastroenterology and Nutrition*.

[10] Mollayeva T., Thurairajah P., Burton K., Mollayeva S., Shapiro C. M., Colantonio A. (2016). The Pittsburgh sleep quality index as a screening tool for sleep dysfunction in clinical and non-clinical samples: a systematic review and meta-analysis. *Sleep Medicine Reviews*.

[11] Buysse D. J., Reynolds C. F., Monk T. H., Berman S. R., Kupfer D. J. (1989). The Pittsburgh Sleep Quality Index: a new instrument for psychiatric practice and research. *Psychiatry Research*.

[12] Passos M. H. P., Silva H. A., Pitangui A. C. R., Oliveira V. M. A., Lima A. S., Araújo R. C. (2017). Confiabilidade e validade da versao brasileira do Índice de Qualidade do Sono de Pittsburgh em adolescentes. *Jornal de Pediatria*.

[13] Sen T., Spruyt K. (2020). Pediatric sleep tools: an updated literature Review. *Frontiers in Psychiatry*.

[14] Savedra M. C., Holzemer W. L., Tesler M. D., Wilkie D. J. (1993). Assessment of postoperation pain in children and adolescents using the adolescent pediatric pain tool. *Nursing Research*.

[15] Walker L. S., Greene J. W. (1991). The functional disability inventory: measuring a neglected dimension of child health status. *Journal of Pediatric Psychology*.

[16] Kashikar-Zuck S., Flowers S. R., Claar R. L. (2011). Clinical utility and validity of the Functional Disability Inventory among a multicenter sample of youth with chronic pain. *Pain*.

[17] Chorpita B. F., Yim L., Moffitt C., Umemoto L. A., Francis S. E. (2000). Assessment of symptoms of DSM-IV anxiety and depression in children: a revised child anxiety and depression scale. *Behaviour Research and Therapy*.

[18] Chorpita B. F., Moffitt C. E., Gray J. (2005). Psychometric properties of the Revised Child Anxiety and Depression Scale in a clinical sample. *Behaviour Research and Therapy*.

[19] Field A. (2009). *Discovering Statistics Using SPSS*.

[20] Cole J. C., Motivala S. J., Buysse D. J., Oxman M. N., Levin M. J., Irwin M. R. (2006). Validation of a 3-factor scoring model for the Pittsburgh Sleep Quality Index in older adults. *Sleep*.

[21] Stevens J. (1996). *Applied Multivariate Statistics for the Social Sciences*.

[22] Kaiser H. F. (1960). The application of electronic computers to factor analysis. *Educational and Psychological Measurement*.

[23] Hair J. F., Black W. C., Babin B. J., Anderson R. E. (2014). *Multivariate Data Analysis*.

[24] Yong A. G., Pearce S. (2013). A beginners guide to factor analysis: focusing on exploratory factor analysis. *Tutorials in quantitative methods for psychology*.

[25] Bollen K. A., Long J. S. (1993). *Testing Structural Equation Models*.

[26] Hoyle R. H. (1995). *Structural Equation Modeling: Concepts, Issues, and Applications*.

[27] Kline R. B. (2005). *Principles and Practice of Structural Equation Modeling, 2nd Ed*.

[28] Bentler P. M., Bonett D. G. (1980). Significance tests and goodness of fit in the analysis of covariance structures. *Psychological Bulletin*.

[29] Marsh H. W., Hocevar D. (1985). Application of confirmatory factor analysis to the study of self-concept: first- and higher order factor models and their invariance across groups. *Psychological Bulletin*.

[30] Browne M. W., Cudeck R., Bollen K. A., Long J. S. (1993). *Alternative ways of assessing model fit*.

[31] Hu L., Bentler P. M. (1999). Cutoff criteria for fit indexes in covariance structure analysis: conventional criteria versus new alternatives. *Structural Equation Modeling: A Multidisciplinary Journal*.

[32] Burnham K. P., Anderson D. R. (1998). *Model Selection and Inference: A Practical Information-Theoretic Approach*.

[33] Akaike H. (1974). A new look at the statistical model identification. *IEEE Transactions on Automatic Control*.

[34] Schreiber J. B., Nora A., Stage F. K., Barlow E. A., King J. (2006). Reporting structural equation modeling and confirmatory factor analysis results: a review. *The Journal of Educational Research*.

[35] Manzar M. D., BaHammam A. S., Hameed U. A. (2018). Dimensionality of the Pittsburgh Sleep Quality Index: a systematic review. *Health and Quality of Life Outcomes*.

[36] Tarokh L., Saletin J. M., Carskadon M. A. (2016). Sleep in adolescence: physiology, cognition and mental health. *Neuroscience and Biobehavioral Reviews*.

[37] Evans S., Djilas V., Seidman L. C., Zeltzer L. K., Tsao J. C. I. (2017). Sleep quality, affect, pain, and disability in children with chronic pain: is affect a mediator or moderator?. *The Journal of Pain: Official Journal of the American Pain Society*.

[38] Shelgikar A. V., Chervin R. (2013). Approach to and evaluation of sleep disorders. *CONTINUUM: Lifelong Learning in Neurology*.

